# The Progression Patterns and Subsequent Treatments of First‐Line Immunotherapy in Advanced Non‐Small Cell Lung Cancer: A Retrospective Cohort Study

**DOI:** 10.1111/1759-7714.70173

**Published:** 2025-10-15

**Authors:** Qi He, Xiao‐bei Guo, Yu‐rou Chen, Xiao‐xing Gao, Min‐jiang Chen, Jing Zhao, Wei Zhong, Yan Xu, Meng‐zhao Wang

**Affiliations:** ^1^ Department of Respiratory and Critical Care Medicine Peking Union Medical College Hospital, Chinese Academy of Medical Sciences and Peking Union Medical College Beijing China

**Keywords:** immune checkpoint inhibitors, non‐small cell lung cancer, oligoprogression, patterns of progression, systemic progression

## Abstract

**Background:**

In metastatic non‐small cell lung cancer (NSCLC) patients receiving first‐line immune checkpoint inhibitors (ICIs), the real‐world progression patterns and subsequent treatment outcomes remain controversial.

**Methods:**

We retrospectively analyzed patients with stage IV NSCLCs treated with first‐line immunotherapy between January 2017 and June 2023. Clinico‐demographic and treatment data were obtained from an electronic medical record system. Progression patterns were categorized by: (1) Progression in different organs; (2) Progression in existing or new lesions; and (3) Oligoprogression versus systemic progression. Survival was assessed using the Kaplan–Meier method and Cox proportional hazards models.

**Results:**

Among 157 patients, 67.5% experienced systemic progression, and 49.0% had progression in existing lesions. The most common organs with progression were the lung (81.5%), lymph nodes (35.0%), and pleural effusion (24.2%). Median post‐progression survival (PPS) was superior in oligoprogression versus systemic progression (22.4 vs. 10.9 months, *p* = 0.012). Patients with extrapulmonary progression (8.2 vs. 22.9 months, *p* < 0.001) or progression in new lesions (9.6 vs. 25.3 months, *p* = 0.029) showed decreased PPS. In multivariate Cox regression, ECOG PS of 2–4 (HR: 2.26, *p* = 0.003), tumor stage of IVB (HR: 1.74, *p* = 0.013), and extrapulmonary progression (HR: 2.05, *p* = 0.002) were associated with decreased PPS. Subsequent treatments containing ICIs improved survival compared to regimens without ICIs (29.0 vs. 11.3 months, *p* = 0.004), particularly in patients with systemic progression (19.3 vs. 9.2 months, *p* = 0.013).

**Conclusion:**

Most metastatic NSCLCs on first‐line immunotherapy experienced systemic progression. Oligoprogression and progression only in existing lesions showed better prognoses, whereas extrapulmonary progression indicated worse survival. Subsequent ICI‐containing treatments had improved survival.

## Graphical abstract

In patients with advanced NSCLCs receiving first‐line immunotherapy, most experienced systemic progression and had progression in existing lesions. Extrapulmonary progression was associated with decreased post‐progression survival, whereas oligoprogression and progression only in existing lesions showed better prognoses. After progression on first‐line immunotherapy, subsequent treatments with immunotherapy had improved survival, particularly in patients with systemic progression and acquired resistance.

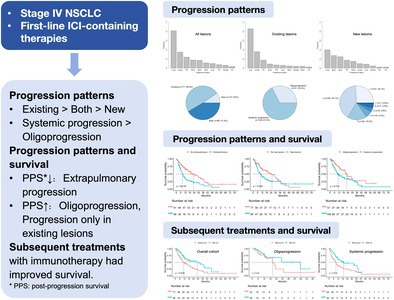

## 1 Introduction

Lung cancer is the most commonly diagnosed cancer and the leading cause of cancer‐related mortality worldwide [1]. Non‐small cell lung cancer (NSCLC) is the most common pathological subtype [2]. In recent years, immune checkpoint inhibitors (ICIs), including anti‐PD‐1 and anti‐PD‐L1 inhibitors, have significantly improved the survival of patients with metastatic NSCLC. As a result, ICI monotherapy and immunochemotherapy have become the first‐line treatments for metastatic NSCLC without driver mutations [3].

However, most advanced NSCLCs eventually develop resistance to ICIs and exhibit disease progression [4]. Resistance to ICIs is typically classified as primary and acquired resistance, depending on the time to progression [5]. Primary resistance refers to patients receiving immunotherapy and developing progression disease (PD) within 6 months [4]. In contrast, acquired resistance occurs when patients with NSCLC initially respond to ICIs but relapse after 6 months and develop PD [4].

The progression patterns after first‐line immunotherapy are crucial for predicting patient prognosis and selecting subsequent treatment strategies. Progression patterns include oligoprogression and systemic progression based on the number of progressive organs or lesions [6]. Oligoprogression occurs in a limited number of organs or lesions, usually within 3–5 lesions in 1–3 organs, and can be treated with local therapies [7, 8]. For oligoprogression, the treatment strategy is to use radiotherapy, surgery, or other local therapies to treat progressive organs and continue systemic ICI treatment [6]. Radiotherapy can control progressive lesions and plausibly contribute to the re‐sensitization of the tumor to immunotherapy [9]. However, systemic progression is widespread in multiple organs and cannot be promptly controlled using local therapies [7, 10]. It is defined as progression in more than three to five organs or lesions [11, 12, 13]. Treatment strategies for systemic progression included chemotherapy, anti‐vascular therapies, ICI rechallenge, ICI‐based combinations with chemotherapy or anti‐vascular agents, or other novel anti‐cancer agents [6, 10, 11].

Although some studies have evaluated progression patterns of metastatic NSCLC after immunotherapy, several issues remain unresolved. The definition of oligoprogression varied among studies, which led to an inconsistent occurrence of oligoprogression, ranging from 36.2% to 82.5% [8, 11, 12, 13, 14, 15, 16]. In addition, previous studies have not reached a consensus on subsequent treatment regimens for systemic progression, and the efficacy of second‐line treatments was not satisfying [17]. While some studies have suggested that anti‐vascular therapies could provide survival benefits for this cohort, others indicated that immunotherapy combined with other agents improved survival outcomes [11, 14, 15]. The heterogeneity of the outcome definitions, treatment lines, and patient characteristics may explain the variation among the studies. Therefore, further research is required to elucidate the progression patterns of metastatic NSCLC after immunotherapy, focusing on when progression occurs, the organs affected by progression, and the outcomes of different subsequent treatments.

In this study, we retrospectively analyzed the data of patients with metastatic NSCLC treated with first‐line immunotherapy. This study aimed to delineate the progression patterns of first‐line ICI‐based treatments and the survival outcomes of patients with different progression patterns. We also investigated subsequent regimens in real‐world scenarios, which provided information for selecting treatment strategies after the failure of first‐line immunotherapy in clinical practice.

## 2 Methods

### 2.1 Participants

Patients with stage IV NSCLC who underwent first‐line immunotherapy between January 2017 and June 2023 were enrolled at the Peking Union Medical College Hospital (PUMCH). Data were retrospectively collected from the electronic medical records database of PUMCH. This study was conducted in accordance with the Declaration of Helsinki and approved by the Ethics Committee of PUMCH (Beijing, China, Ethics Number: JS‐1410).

The inclusion criteria were as follows: (1) pathologically diagnosed NSCLC; (2) clinical stage IV based on the TNM staging system (8th edition, International Association for the Study of Lung Cancer); (3) treatment with first‐line anti‐PD‐1 or anti‐PD‐L1 ICI monotherapy or in combination with chemotherapy or anti‐vascular agents; and (4) follow‐up for at least 30 days. The exclusion criteria were as follows: (1) with EGFR, ALK, or ROS1 gene alterations; (2) without measurable lesions according to the Response Evaluation Criteria in Solid Tumors (RECIST) version 1.1; (3) without response assessment during immunotherapy treatment; (4) without available chest computed tomography (CT), brain magnetic resonance imaging (MRI), bone scan, and/or positron emission tomography (PET)/CT before initiating immunotherapy or during follow‐up. Patients with radiologically confirmed PD were included in the final analysis.

### 2.2 Data Collection

Based on the electronic medical record system, we collected demographic and clinical data, including sex, age, smoking history, Eastern Tumor Cooperation Group performance status (ECOG PS), TNM stage, tumor pathology, PD‐L1 expression, first‐line treatment regimens, baseline metastatic organs, number of progressions, subsequent treatment regimens, and adverse events. Survival data were obtained from the CAPTRA‐LUNG database during regular follow‐ups through inpatient visits, hospitalizations, and telephone calls [18]. The follow‐up cutoff date was January 2025.

### 2.3 Definitions of Outcomes

According to RECIST version 1.1, the response to first‐line and subsequent treatments was assessed by the best overall response and classified as complete response (CR), partial response (PR), stable disease (SD), or progression disease (PD). The objective response rate (ORR) was defined as the proportion of patients with CR and PR, whereas the disease control rate (DCR) was defined as the proportion of patients with CR, PR, or SD as the best overall response.

Based on the number of progressive organs or lesions, PD was further divided into oligoprogression and systemic progression. In this study, oligoprogression was progression in not more than three organs and no more than three lesions that could be treated by local therapies, including multiple progressive lesions in the brain that could be treated by local therapies. Systemic progression was progression in at least three organs or lesions, including progression of pleural effusion, pericardial effusion, malignant ascites, and leptomeningeal disease. Acquired resistance (AR) was defined as PD after more than 6 months of initial response to immunotherapy. Primary resistance was defined as PD within 6 months after initiating first‐line immunotherapy.

For survival outcomes, the first progression‐free survival (PFS1) was defined as the time between the first cycle of first‐line immunotherapy and the first documented PD or last follow‐up. The second PFS (PFS2) was defined as the time from the first PD to the second PD or last follow‐up. The overall survival (OS) was defined as the time from the first course of first‐line immunotherapy to death from any cause or the last follow‐up. The post‐progression survival (PPS) was defined as the time from the first PD to death from any cause or last follow‐up. The immune‐related adverse events (irAEs) were evaluated according to CTCAE version 5.0.

### 2.4 Statistical Analysis

Continuous variables were described as mean (or median) and standard deviation (or range), while categorical variables were described as count and percentage. Differences of continuous variables were assessed by *t*‐tests, whereas Chi‐square or Fisher's exact tests were applied for categorical variables. The Kaplan–Meier method was used to calculate PFS1, PFS2, OS, and PPS, and the log‐rank test was applied to compare survival outcomes between different groups. The univariate and multivariate Cox proportional hazard models were applied to estimate the association between patient characteristics and clinical outcomes. The hazard ratios (HR) and 95% confidence intervals (CI) were calculated. Statistical tests were two‐sided, and *p* < 0.05 was considered statistically significant. All statistical analyses were performed using R software (version 4.3.1).

## 3 Results

### 3.1 Patient Characteristics

**FIGURE 1:**
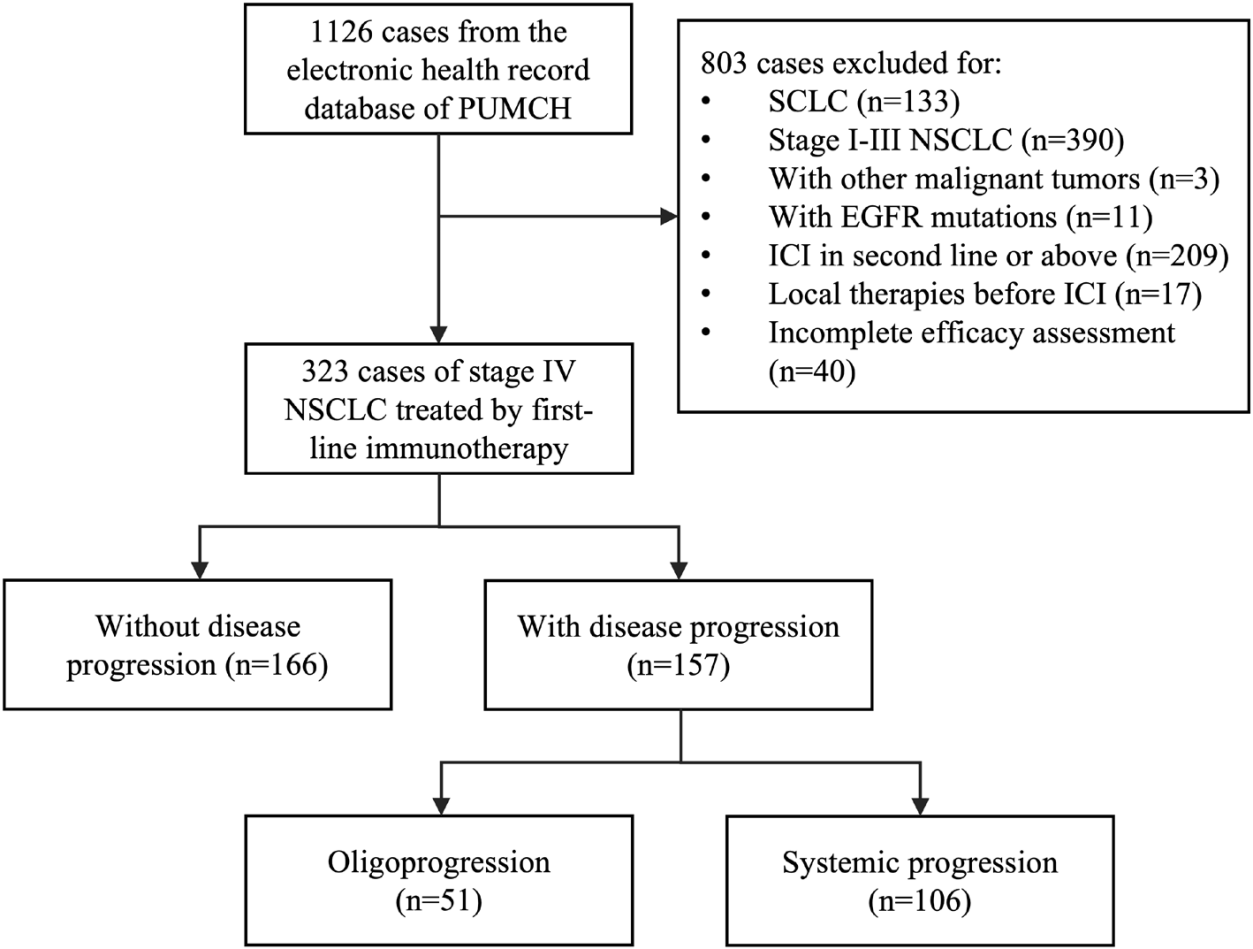
Flow diagram of the study.

A total of 157 patients with advanced NSCLC developed PD on first‐line immunotherapy. The flow diagram for patient selection is shown in Figure 1, and the clinical characteristics at first disease progression are presented in Table 1. In the total population, 59.9% (94/157) of patients died, and the median follow‐up duration was 35.9 (95% CI: 31.3–46.4) months. The median age was 65 years (range, 30–88 years), and 77.7% (122/157) were male. Most patients had a smoking history (111/157, 70.7%) and had an ECOG performance score of 0 to 1 (134/157, 85.4%). The pathology of tumors was most commonly adenocarcinoma (91/157, 58.0%), and the percentage of patients with PD‐L1 expression of < 1%, 1%–49%, and ≥ 50% was 14.6% (23/157), 22.3% (35/157), and 14.0% (22/157), respectively. As for treatment regimens, the majority of patients received ICI combined with chemotherapy (139/157, 88.5%), and the median cycles of immunotherapy were 8 (range, 1–49). The ICI agents included anti‐PD‐1 (151/157, 96.2%), anti‐PD‐L1 (3/157, 1.9%), anti‐PD‐1 plus anti‐CTLA‐4 (2/157, 1.3%), and others (1/157, 0.6%).

**TABLE 1:** Patient and tumor characteristics at first progression disease.

Characteristics	Total (*n* = 157)
Age	65 (30–88)
Sex	
Male	122 (77.7)
Female	35 (22.3)
Smoking status	
No smoking history	46 (29.3)
Current smoker	51 (32.5)
Former smoker	60 (38.2)
ECOG PS	
0	66 (42.0)
1	68 (43.3)
2–4	23 (14.6)
Stage of tumor	
IVA	79 (50.3)
IVB	78 (49.7)
Histology of tumor	
Adenocarcinoma	91 (58.0)
Squamous	62 (39.5)
Other	4 (2.5)
PD‐L1 expression	
< 1%	23 (14.6)
1%–49%	35 (22.3)
≥ 50%	22 (14.0)
Not available	77 (49.0)
Type of immunotherapy	
ICI monotherapy	18 (11.5)
ICI + chemotherapy	139 (88.5)
ICI agents	
Anti‐PD‐1	151 (96.2)
Anti‐PD‐L1	3 (1.9)
Anti‐PD‐1 + Anti‐CTLA‐4	2 (1.3)
Others	1 (0.6)
Cycles of immunotherapy	8 (1–49)
Best overall response	
PR	71 (45.2)
SD	73 (46.5)
PD	13 (8.3)

*Note:* Age is described as median (range), and other variables are described as the number (percentage, %).

Abbreviations: NE, not estimable; Squamous, squamous cell carcinoma.

Overall, the ORR for first‐line immunotherapy was 45.2%, and the DCR was 91.7%. The median PFS for first‐line immunotherapy was 7.6 (95% CI: 6.4–9.4) months, and the median OS was 28.4 (95% CI: 22.3–37.5) months (Figure S1). As for safety, 35.7% (56/157) of patients experienced all‐grade irAEs, and 10.2% (16/157) had grade 3 or above irAEs (Table S1).

### 3.2 Progression Patterns

**FIGURE 2:**
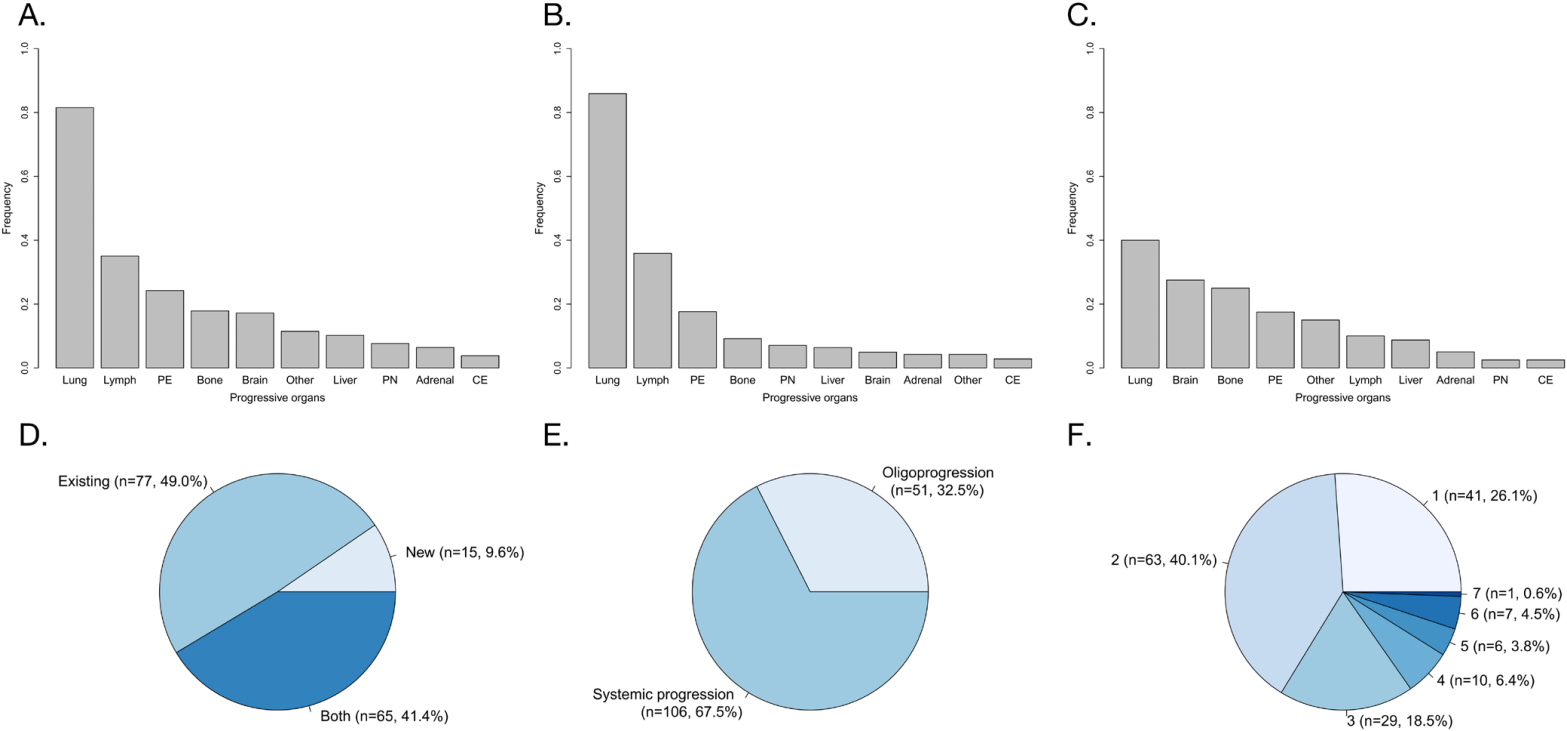
Progression patterns of first‐line immunotherapy in metastatic NSCLC. (A) Progression organs in all lesions; (B) progression organs in existing lesions; (C) progression organs in new lesions; (D) progression in new, existing, or both new and existing lesions; (E) oligoprogression or systemic progression; (F) number of progression organs. CE, pericardial effusion; PE, pleural effusion; PN, pleural nodules.

The progression patterns are presented in Figure 2. In this study, we used progression‐free survival (PFS1) as an alternative indicator for resistance types, with PFS1 ≥ 6 months defined as acquired resistance, and PFS1 < 6 months defined as primary resistance. Under this definition, 59.9% (94/157) of patients had acquired resistance to first‐line immunotherapy, whereas others (63/157, 40.1%) developed primary resistance.

As for organs with progression, the most common organs were the lung (128/157, 81.5%), lymph nodes (55/157, 35.0%), and pleural effusion (38/157, 24.2%) (Figure 2A). Specifically, 42.0% (66/157) of patients experienced extrapulmonary progression, and 17.8% (28/157) experienced intracranial progression.

Regarding progression in existing or new lesions, the progression solely in existing lesions was found in 49.0% (77/157) of patients, whereas 9.6% (15/157) developed progression in new lesions only, and 41.4% (65/157) developed progression in both new and existing lesions (Figure 2D). Among 142 patients with progression in existing lesions, the most common organs were the lung (122/142, 85.9%), lymph nodes (51/142, 35.9%), pleural effusion (25/142, 17.6%), and bone (13/142, 9.2%) (Figure 2B). On the other hand, among the 80 patients with progression in new lesions, the most common organs were the lung (32/80, 40.0%), brain (22/80, 27.5%), bone (20/80, 25.0%), and pleural effusion (14/80, 17.5%) (Figure 2C).

In terms of the number of progressive organs or lesions, 32.5% (51/157) of patients developed oligoprogression, and 67.5% (106/157) developed systemic progression (Figure 2E). The number of progressive organs was as follows: one organ in 26.1% (41/157), two organs in 40.1% (63/157), three organs in 18.5% (29/157), four organs in 6.4% (10/157), five organs in 3.8% (6/157), and ≥ 6 organs in 5.1% (8/157) (Figure 2F).

### 3.3 Survival

**FIGURE 3:**
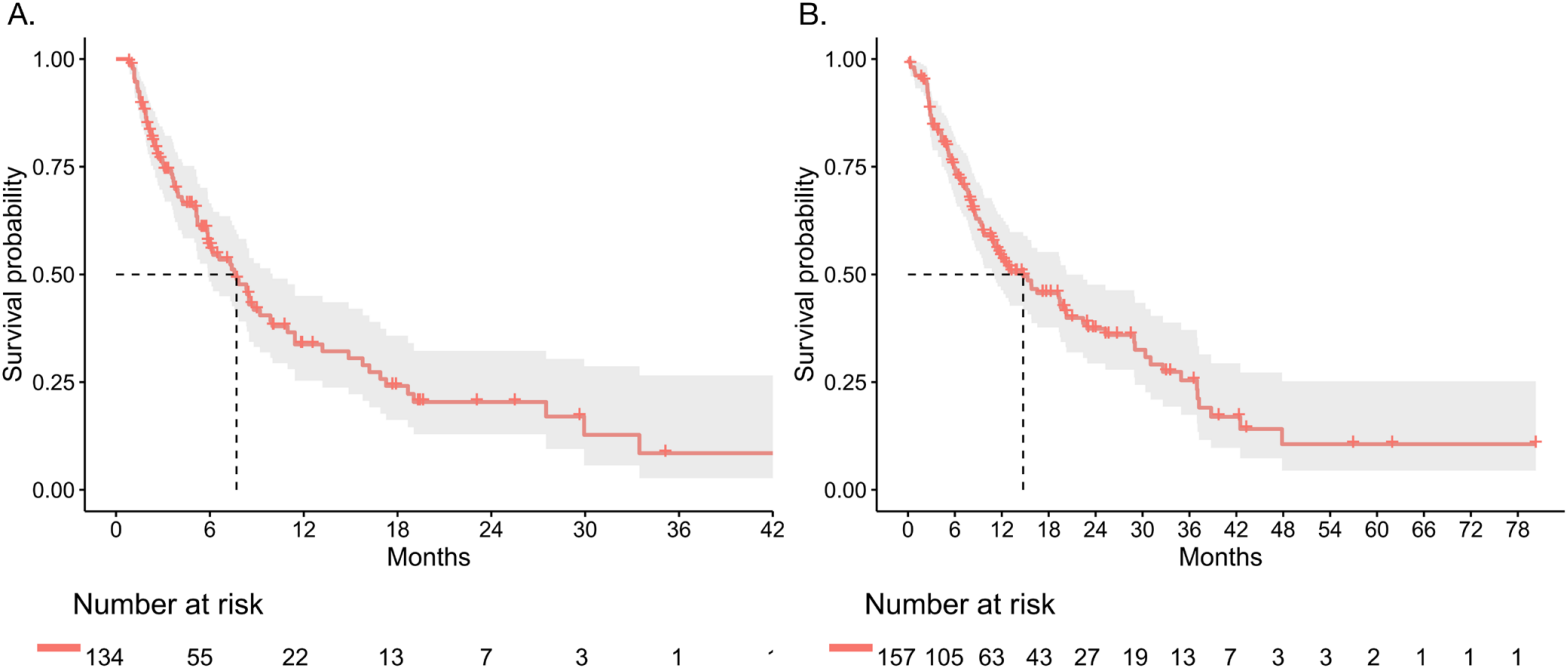
Survival of the total population after progression on first‐line immunotherapy. (A) PFS2 (median: 7.7 months, 95% CI: 5.9–10.0 months); (B) PPS (median: 14.7 months, 95% CI: 10.9–20.3 months).

After progression on the first‐line immunotherapy, the median PFS2 of the total population was 7.7 months (95% CI: 5.9–10.0 months), and the median PPS was 14.7 months (95% CI: 10.9–20.3 months) (Figure 3).

**FIGURE 4:**
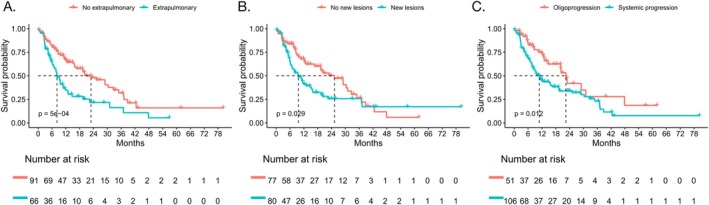
Survival stratified by different progression patterns. (A) PPS in patients with or without extrapulmonary progression (8.2 vs. 22.9 months, *p* < 0.001); (B) PPS in patients without or with new lesions (9.6 vs. 25.3 months, *p* = 0.029); (C) PPS in patients with systemic progression or oligoprogression (10.9 vs. 22.4 months, *p* = 0.012).

Survival outcomes varied across different progression patterns (Figure 4). Patients with extrapulmonary progression had decreased PPS compared to patients without extrapulmonary progression (median: 8.2 vs. 22.9 months, *p* < 0.001) (Figure 4A). Similarly, for progression in existing or new lesions, the PPS was decreased in patients with progression in new lesions than in those with progression only in existing lesions (median: 9.6 vs. 25.3 months, *p* = 0.029) (Figure 4B). In addition, patients with oligoprogression showed significantly improved PPS compared to systemic progression (median: 22.4 vs. 10.9 months, *p* = 0.012) (Figure 4C). On the other hand, PFS2 did not show significant differences between patients without or with extrapulmonary progression (8.4 vs. 5.9 months, *p* = 0.363), progression in existing or new lesions (9.2 vs. 5.9 months, *p* = 0.199), and oligoprogression versus systemic progression (8.4 vs. 6.6 months, *p* = 0.791).

Therefore, after the first PD on first‐line immunotherapy, post‐progression survival differed for varied progression patterns. Extrapulmonary progression, progression in new lesions, and systemic progression were associated with worse PPS, but the difference was not significant for PFS2.

### 3.4 The Relationship Between Clinical Factors, Progression Patterns, and Overall Survival

To further analyze the association between progression patterns and survival, the univariate and multivariate Cox regression were performed with clinical factors as covariates. In univariate analysis, ECOG PS of 2–4 (HR: 2.76, 95% CI: 1.65–4.62, *p* < 0.001), tumor stage of IVB (HR: 2.18, 95% CI: 1.43–3.32, *p* < 0.001), best overall response of SD or PD (HR: 1.77, 95% CI: 1.17–2.68, *p* = 0.007), extrapulmonary progression (HR: 2.05, 95% CI: 1.36–3.09, *p* < 0.001), progression in new lesions (HR: 1.57, 95% CI: 1.04–2.37, *p* = 0.031), and systemic progression (HR: 1.84, 95% CI: 1.14–2.97, *p* = 0.013) were associated with worse PPS (Table 2). In multivariate analysis, the following variables remained significant: ECOG PS of 2–4 (HR: 2.26, 95% CI: 1.33–3.84, *p* = 0.003), tumor stage of IVB (HR: 1.74, 95% CI: 1.12–2.71, *p* = 0.013), and extrapulmonary progression (HR: 2.05, 95% CI: 1.29–3.25, *p* = 0.002). Progression in new lesions (HR: 1.15, 95% CI: 0.73–1.81, *p* = 0.540) and systemic progression (HR: 1.51, 95% CI: 0.88–2.57, *p* = 0.133) were not significant in the multivariate model.

**TABLE 2:** Univariate and multivariate Cox regression analysis of post‐progression survival and characteristics at first PD.

Characteristics	Total (*n* = 157)	PPS (months, 95% CI)	Univariate analysis		Multivariate analysis	
			HR (95% CI)	*p*	HR (95% CI)	*p*
Age						
≥ 65	82 (52.2)	15.8 (9.2–22.9)	1.22 (0.81–1.84)	0.339		
< 65	75 (47.8)	14.7 (10.9–32.6)				
Sex						
Female	35 (22.3)	20.2 (9.6–NE)	1.22 (0.74–2.02)	0.444		
Male	122 (77.7)	13.0 (9.8–20.3)				
Smoking status						
No smoking history	46 (29.3)	20.2 (11.3–NE)	1.39 (0.87–2.22)	0.175		
Smoking history	111 (70.7)	12.5 (9.4–20.3)				
ECOG PS						
0–1	134 (85.4)	19.3 (12.5–28.9)	2.76 (1.65–4.62)	**< 0.001**	2.26 (1.33–3.84)	**0.003**
2–4	23 (14.6)	5.1 (3.1–11.3)				
Stage of tumor						
IVA	79 (50.3)	20.3 (15.8–42.5)	2.18 (1.43–3.32)	**< 0.001**	1.74 (1.12–2.71)	**0.013**
IVB	78 (49.7)	8.6 (6.7–12.5)				
Histology of tumor						
Adenocarcinoma	91 (58.0)	11.5 (8.7–29.0)	0.84 (0.56–1.27)	0.404		
Squamous and other	66 (42.0)	19.3 (12.5–30.4)				
PD‐L1 expression						
< 1%	23 (14.6)	19.4 (10.9–NE)	0.81 (0.43–1.50)	0.496		
≥ 1%	57 (36.3)	20.3 (14.7–37.2)				
Type of immunotherapy						
ICI monotherapy	18 (11.5)	12.2 (7.7–NE)	1.10 (0.60–2.04)	0.758		
ICI + chemotherapy	139 (88.5)	14.7 (10.9–25.3)				
Best overall response						
PR	71 (45.2)	19.5 (14.7–37.2)	1.77 (1.17–2.68)	**0.007**	1.39 (0.88–2.18)	0.159
SD or PD	86 (54.8)	9.2 (7.7–20.2)				
Extrapulmonary progression						
No	91 (58.0)	22.9 (15.8–34.9)	2.05 (1.36–3.09)	**< 0.001**	2.05 (1.29–3.25)	**0.002**
Yes	66 (42.0)	8.2 (6.7–12.9)				
New lesion progression						
No	77 (49.0)	25.3 (16.5–32.6)	1.57 (1.04–2.37)	**0.031**	1.15 (0.73–1.81)	0.540
Yes	80 (51.0)	9.6 (6.8–15.3)				
Oligoprogression or systemic progression						
Oligoprogression	51 (32.5)	22.4 (14.7–NE)	1.84 (1.14–2.97)	**0.013**	1.51 (0.88–2.57)	0.133
Systemic progression	106 (67.5)	10.9 (8.1–16.5)				

*Note:* Bold indicates statistically significant results at *p* < 0.05.

Abbreviation: NE, not estimable.

### 3.5 Subsequent Treatments After Progression

Subsequent treatments after PD are shown in Table 3 and Figure S2. Among the total population, 94.4% (135/143) of patients received systemic therapies, and 24.5% (35/143) received local therapies. Among the patients receiving local therapies, the most common treatment was radiotherapy (30/143, 21.0%), followed by surgery (5/143, 3.5%), radiofrequency ablation (2/143, 1.4%), and other local therapies (1/143, 0.7%). The sites of radiotherapy included the brain (14/30, 46.7%), lungs (8/30, 26.7%), bones (6/30, 20.0%), distant lymph nodes (2/30, 6.7%), and pancreas (2/30, 6.7%).

**TABLE 3:** Treatment strategies after progression on first‐line immunotherapy.

Subsequent treatments	Total (*n* = 143)	Oligoprogression (*n* = 49)	Systemic progression (*n* = 94)	*p*
Local therapies	**35 (24.5)**	**20 (40.8)**	**15 (16.0)**	0.002
Radiotherapy	30 (21.0)	16 (32.7)	14 (14.9)	0.024
Ablation	2 (1.4)	2 (4.1)	0 (0.0)	1
Surgery	5 (3.5)	2 (4.1)	3 (3.2)	1
Other	1 (0.7)	1 (2.0)	0 (0.0)	1
Systemic therapies	**135 (94.4)**	**42 (85.7)**	**93 (98.9)**	1
Chemotherapy	101 (70.6)	27 (55.1)	74 (78.7)	0.006
Antivascular therapies	38 (28.1)	10 (23.8)	28 (30.1)	0.315
Target therapies	1 (0.7)	0 (0.0)	1 (1.1)	1
ICI	66 (46.2)	26 (53.1)	40 (40.4)	0.308

*Note:* Bold indicates statistically significant results at *p* < 0.05. Variables were described as number (percentage, %).

The most common systemic therapies were chemotherapy (101/143, 70.6%) and ICI (66/143, 46.2%), whereas others included anti‐vascular therapies (38/143, 28.1%) and target therapies (1/143, 0.7%). Specifically, subsequent chemotherapy included platinum doublet chemotherapy (39/101, 38.6%) and single‐agent chemotherapy (62/101, 61.4%). Subsequent anti‐vascular therapies included anlotinib (20/38, 52.6%), bevacizumab (14/38, 36.8%), and other anti‐vascular agents (5/38, 13.2%). Subsequent immunotherapy included maintaining first‐line ICIs (57/66, 86.4%) or switching to other ICIs (9/66, 13.6%). One patient received HER2‐targeting therapy (DS8201, T‐Dxd) after the first PD.

Subsequent treatments varied for patients with oligoprogression and systemic progression (Table 3). Local therapies, mostly radiotherapy, were more commonly found in patients with oligoprogression than in those with systemic progression (40.8% vs. 16.0%, *p* = 0.002). Similarly, ICIs were numerically more common in patients with oligoprogression (oligo vs. systemic: 53.1% vs. 40.4%, *p* = 0.308). On the other hand, a higher proportion of patients received chemotherapy in those with systemic progression than in those with oligoprogression (78.7% vs. 55.1%, *p* = 0.006) as subsequent therapies.

For combinations of systemic treatments, the commonly applied regimens included chemotherapy alone (41/143, 28.7%), chemotherapy plus immunotherapy (40/143, 28.0%), ICI alone (16/143, 11.2%), and anti‐vascular therapy alone (16/143, 11.2%) (Table 4).

**TABLE 4:** Subsequent treatments and survival.

Subsequent treatments	Total (*n* = 143)	Number of death	OS (months, 95% CI)
ICI (+Local)	16	8	47.8 (8.6–NE)
ICI + Chemotherapy (+Local)	40	14	32.6 (15.8–NE)
ICI + Chemotherapy + Anti‐vascular (+Local)	8	6	13.6 (6.7–NE)
ICI + Anti‐vascular (+Local)	2	1	37.2 (NE–NE)
Chemotherapy (+Local)	41	31	7.5 (5.2–19.5)
Chemotherapy + Anti‐vascular (+Local)	12	10	12.2 (10.9–NE)
Anti‐vascular	16	10	7.7 (2.8–NE)
Local therapy only	7	2	31.0 (22.9–NE)
Target therapy	1	0	NE

Abbreviations: Anti‐vascular, anti‐vascular therapies; ICI, immune checkpoint inhibitor; Local, local therapies; NE, not estimable.

**FIGURE 5:**
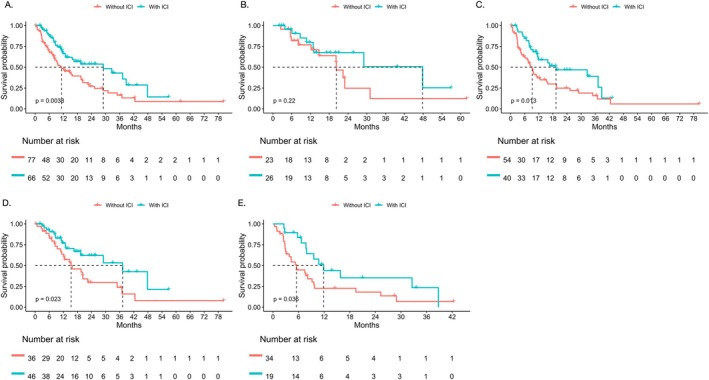
Survival of different subsequent treatments with or without ICIs. (A) PPS of patients receiving subsequent treatments with or without ICIs (29.0 vs. 11.3 months, *p* = 0.004); (B) PPS of patients with oligoprogression and receiving subsequent treatments with or without ICIs (47.8 vs. 20.3 months, *p* = 0.222); (C) PPS of patients with systemic progression and receiving subsequent with or without ICIs (19.3 vs. 9.2 months, *p* = 0.013); (D) PPS of patients with acquired resistance and receiving subsequent treatments with or without ICIs (37.2 vs. 15.3 months, *p* = 0.023); (E) PPS of patients with primary resistance and receiving subsequent treatments with or without ICIs (11.9 vs. 5.5 months, *p* = 0.036).

Subsequent regimens containing ICIs showed survival benefits (Figure 5). For the overall cohort, treatment regimens with ICIs had significantly prolonged PPS compared to regimens without ICIs (29.0 vs. 11.3 months, *p* = 0.004) (Figure 5A). Similarly, in patients with systemic progression, regimens with ICIs also showed the benefit of PPS (19.3 vs. 9.2 months, *p* = 0.013) (Figure 5C). However, for patients with oligoprogression, the PPS showed a trend of benefit in patients receiving ICIs, but the difference was not statistically significant (47.8 vs. 20.3 months, *p* = 0.222) (Figure 5B). On the other hand, PFS2 was similar in patients receiving subsequent regimens with or without ICIs in the overall cohort (7.4 vs. 7.7 months, *p* = 0.550), patients with oligoprogression (9.2 vs. 7.7 months, *p* = 0.546), and patients with systemic progression (5.9 vs. 7.3 months, *p* = 0.979).

For patients who developed acquired resistance to first‐line immunotherapy, those receiving subsequent treatments with ICIs had significantly improved PPS than those without ICI treatments (37.2 vs. 15.3 months, *p* = 0.023) (Figure 5D). Also, adding ICIs was associated with increased PPS in patients with primary resistance (11.9 vs. 5.5 months, *p* = 0.036) (Figure 5E). On the other hand, in both acquired resistance and primary resistance cohorts, PFS2 did not show significant differences for patients receiving or not receiving subsequent ICIs (Acquired resistance: 6.2 vs. 8.5 months, *p* = 0.639; Primary resistance: 8.8 vs. 6.6 months, *p* = 0.320).

Therefore, subsequent treatments with ICIs were associated with increased PPS but not PFS2, particularly in patients with systemic progression.

## 4 Discussion

In this study, we retrospectively analyzed the progression patterns of 157 patients with advanced NSCLC who received first‐line immunotherapy. Most patients experienced systemic progression and had progression to the existing lesions. The most common organs of progression were the lungs, lymph nodes, and pleural effusion, and new lesions were more commonly found in the lungs, brain, and bones. Patients with oligoprogression, or progression only in existing lesions, had prolonged survival. On the other hand, extrapulmonary progression was associated with worse survival and served as a predictor for worse PPS in both univariate and multivariate analyses. Subsequent treatments containing ICIs had significantly improved survival compared to regimens without ICIs, particularly for patients with systemic progression or acquired resistance to first‐line immunotherapy.

ICIs have greatly prolonged the survival of patients with NSCLC in the past few years, but many patients developed resistance to immunotherapy during first‐line treatments [19, 20]. The resistance to immunotherapy mainly involves the alterations of lung cancer cells or the tumor microenvironment (TME) [10]. The impairment of tumor immunogenicity, antigen presentation, or mutations in the IFN‐γ pathway could lead to ICI resistance [10, 21]. On the other hand, the change of tumor‐infiltrating lymphocytes can render an immunosuppressive TME and lead to resistance to immunotherapy [4]. For example, T cell exclusion, or dysfunction of T cells, could result in immune resistance [22]. The resistance patterns could be classified as primary or acquired resistance based on time to progression, with the biological mechanisms described as innate or adaptive resistance, respectively [5, 21]. The underlying mechanisms vary for different resistance patterns and can affect the responses to subsequent treatments [4]. Therefore, it is crucial to identify the progression patterns related to prognosis and analyze the optimal combinations for subsequent treatments.

Oligoprogression was progression in only a few organs with well‐controlled metastasis in most lesions and had a better prognosis than systemic progression [23]. Continuing immunotherapy with local radiotherapy or surgery may overcome ICI resistance and improve patients' survival [24]. In advanced NSCLCs receiving ICIs, the incidence of oligoprogression varied among studies. In this cohort, 32.5% of patients developed oligoprogression, and 66.2% had progression in one to two organs. Similarly, in a cohort of 401 patients with PD, Xuzhang et al. [13] found that 36.2% of patients showed oligoprogression. Also, Friedes et al. [8] showed that among 198 patients with PD, 51.5% of patients had no more than three organs of progression, but only 39.9% of patients were classified as having oligoprogression, due to PD in non‐measurable lesions, such as pleural disease. However, other studies suggested that oligoprogression was more common during first‐line immunotherapy. Xu et al. [11] showed that in 208 patients with advanced NSCLC patients who received immunotherapy, 55.3% developed oligoprogression, defined as progression in no more than two organs. Similarly, Chai et al. and Hosoya et al. [12, 25] reported a higher incidence of 58.2% and 70% for oligoprogression, respectively. Therefore, the classification of oligoprogression was mainly based on visible lesions in radiology and might be subject to the physicians' judgments. Thus, future research on progression patterns could combine molecular tests and other biomarkers to identify patient subgroups more accurately.

Consistent with previous studies, our study showed that oligoprogression was associated with significantly prolonged survival compared with systemic progression (median PPS: 22.4 vs. 10.9 months, *p* = 0.012). Additionally, patients with progression only in existing organs had increased PPS compared to patients with progression in new lesions (median PPS: 25.3 vs. 9.6 months, *p* = 0.029). Similarly, Friedes et al. [8] suggested that in advanced NSCLCs receiving first‐line immunotherapy, patients with oligoprogression had significantly longer OS than polyprogression (35.1 vs. 12.2 months, *p* < 0.001). Progression in existing lesions was associated with longer OS than new lesions and both existing and new lesions (28.7 vs. 20.2 vs. 13.9 months, *p* < 0.001). Xu et al. [11] also found that the PFS2 (13.1 vs. 10.0 months, *p* = 0.001) and OS (25.8 vs. 19.1 months, *p* = 0.003) were significantly longer in patients with oligoprogression than systemic progression. Similarly, Schoenfeld et al. [26] suggested that in 143 patients with acquired resistance, oligoprogression was associated with improved overall survival (28 vs. 10 months, *p* < 0.001). Thus, our study supported that oligoprogression and progression only in the existing organs were associated with improved survival.

Our results showed that patients with extrapulmonary progression had worse survival (median PPS: 8.2 vs. 22.9 months, *p* < 0.001), and extrapulmonary progression was associated with decreased PPS in multivariate analysis (HR: 2.05, 95% CI: 1.29–3.25, *p* = 0.002). Previous studies have indicated that baseline bone and brain metastasis at baseline was related to worse survival in patients receiving first‐line immunotherapy [12, 27]. In addition, other research showed that the OS was shortened in patients with progression in distant organs, compared to locoregional progression (locoregional vs. distant vs. both: 39.1 vs. 17.4 vs. 14.3 months, *p* < 0.001) [8]. Therefore, patients with extrapulmonary progression might be indicators of worse survival in subsequent lines of treatment.

Subsequent treatments were also analyzed for different progression patterns. Our study showed that patients with oligoprogression received local therapies and continued ICI treatments more often. In contrast, patients with systemic progression were more likely to be treated with other systemic agents.

Almost half of the patients received regimens containing ICIs as subsequent therapies, which was consistent with several previous studies. Xu et al. [11] analyzed subsequent therapies beyond resistance to immunotherapy, in which 45.7% (95/208) of patients maintained ICI therapy. Similarly, Xuzhang et al. [13] suggested in Supporting Information that 48.6% (195/401) of patients continued ICI therapy after initial immunotherapy failure.

As for the survival of different subsequent therapies, we found that subsequent treatments with ICIs were associated with prolonged post‐progression survival, especially in patients with systemic progression and acquired resistance. Consistent with our results, the BTCRC‐LUN15‐029 trial also suggested that patients with PD receiving ICIs with or without chemotherapy could benefit from subsequent pembrolizumab treatment combined with chemotherapy, compared to single‐agent chemotherapy [28]. A retrospective study by Yu et al. [15] also showed that ICI combination therapies were associated with increased PFS2 and OS compared with other treatment regimens. Thus, continuing ICI or combining ICI with other systemic therapies could reactivate anti‐tumor reactions and provide a survival benefit in patients with PD [29, 30]. Further investigation is needed to identify the characteristics associated with the efficacy of applying ICIs and the optimal treatment combinations after PD on first‐line immunotherapy.

This study had some limitations. First, the single‐center, retrospective nature limited the generalizability. Second, the PD‐L1 expression was not available in nearly 50% of patients, which may affect the explanation of the survival outcomes and progression patterns. Third, the selection of subsequent treatments might be affected by various clinical and social factors, so the association between subsequent regimens and survival after the first PD may be complicated by these confounding factors. Patients with fewer progressive organs or better responses to first‐line ICIs may be more likely to receive second‐line ICIs in real‐world scenarios. Further prospective or randomized studies are required to investigate the effect of adding ICIs in subsequent treatments after the first PD. Lastly, in our cohort, the number of patients receiving subsequent radiotherapy was limited, and the effect of radiotherapy as a subsequent treatment requires further investigation in a larger cohort.

## 5 Conclusions

Patients with advanced NSCLC on first‐line immunotherapy more often experienced systemic progression and had progression in existing lesions. The lungs, lymph nodes, and pleural effusion were the common progressive organs, and the most common organs of new lesions were the lungs, brain, and bones. Oligoprogression and progression only in existing organs had superior prognoses, whereas extrapulmonary progression indicated worse survival. In univariate and multivariate Cox regression analyses, extrapulmonary progression was associated with decreased PPS. Subsequent ICI‐containing treatments had significantly improved OS compared to regimens without ICIs.

## Author Contributions

All authors had full access to the data in the study and take responsibility for the integrity of the data and the accuracy of the data analysis. Conceptualization: M.Z.W., Y.X., and Q.H. Methodology and project administration: Q.H. and X.B.G. Investigation and data curation: Q.H., X.B.G., Y.R.C., X.X.G. Formal analysis: Q.H. Resources: M.J.C., J.Z., W.Z. Writing and original draft: Q.H. Writing – review and editing: M.Z.W., Y.X: Visualization, Q.H. Supervision: M.Z.W., Y.X. Funding acquisition: Y.Z.X.

## Disclosure

The authors have nothing to report.

## Ethics Statement

This study was approved by the Institutional Review Board of Peking Union Medical College Hospital (Ethics Approval Number: JS‐1410).

## Conflicts of Interest

The authors declare no conflicts of interest.

## Supporting information


**Table S1:** Immune‐related adverse events in advanced NSCLC with first‐line immunotherapy.
**Figure S1:** Survival of the total population during first‐line immunotherapy.
(A) PFS1 (median: 7.6 months, 95% CI: 6.4–9.4 months); (B) OS (median: 28.4 months, 95% CI: 22.3–37.5 months).
**Figure S2:** The Sankey diagram of first‐line and second‐line treatment regimens.
Abbreviations: Chemo1, single‐agent chemotherapy; Chemo2, platinum doublet chemotherapy; ICI, immune checkpoint inhibitors; Loc, local therapy; Oligo, oligoprogression; Sys, systemic therapy; Systemic, systemic progression; Target, target therapy; Vasc, anti‐vascular therapy.

## Data Availability

The data that support the findings of this study are available from the corresponding author upon reasonable request.
