# Supplementary material for: The Progression Patterns and Subsequent Treatments of First‐Line Immunotherapy in Advanced Non‐Small Cell Lung Cancer: A Retrospective Cohort Study
Source: Thorac Cancer. 2025 Oct 15;16(20):e70173. doi: 10.1111/1759-7714.70173 (PMC12522518; doi:10.1111/1759-7714.70173)
Supplement: Supplementary file 1 — Table S1: Immune‐related adverse events in advanced NSCLC with first‐line immunotherapy. Figure S1: Survival of the total population during first‐line immunotherapy. (A) PFS1 (median: 7.6 months, 95% CI: 6.4–9.4 months); (B) OS (median: 28.4 months, 95% CI: 22.3–37.5 months). Figure S2: The Sankey diagram of first‐line and second‐line treatment regimens. Abbreviations: Chemo1, single‐agent chemotherapy; Chemo2, platinum doublet chemotherapy; ICI, immune checkpoint inhibitors; Loc, local therapy; Oligo, oligoprogression; Sys, systemic therapy; Systemic, systemic progression; Target, target therapy; Vasc, anti‐vascular therapy. [file TCA-16-e70173-s001.docx]

**Supplementary materials**

**The progression patterns of first-line immunotherapy in advanced non-small cell lung cancer: a retrospective cohort study**

**Authors**: Qi HE ^a^ , Xiao-bei GUO ^a^ , Yu-rou CHEN ^a^ , Xiao-xing GAO ^a^, Min-jiang CHEN ^a^, Jing ZHAO ^a^, Wei ZHONG ^a^, Yan XU ^a^, Meng-zhao WANG ^a †^

**Affiliations**

^a^ Department of Respiratory and Critical Care Medicine, Peking Union Medical College Hospital, Chinese Academy of Medical Sciences and Peking Union Medical College, Beijing, China

**†Correspondence to:**

Mengzhao Wang, MD, PhD, Email: [mengzhaowang@sina.com](mailto:mengzhaowang@sina.com).

Department of Respiratory and Critical Care Medicine, Peking Union Medical College Hospital, Chinese Academy of Medical Sciences & Peking Union Medical College, No. 1 Shuaifuyuan Wangfujing Dongcheng District, Beijing 100730, China.

**Table S1**. Immune-related adverse events in advanced NSCLC with first-line immunotherapy.

| **Immune-related adverse events (irAEs)** | Total  (n=157) | Oligoprogression  (n=51) | Systemic progression  (n=106) |
| --- | --- | --- | --- |
| **All-grade irAEs** | **56 (35.7)** | **22 (43.1)** | **34 (32.1)** |
| Endocrine | 17 (10.8) | 6 (11.8) | 11 (10.4) |
| Skin | 15 (9.6) | 5 (9.8) | 10 (9.4) |
| Respiratory | 8 (5.1) | 5 (9.8) | 3 (2.8) |
| Gastrointestinal | 8 (5.1) | 2 (3.9) | 6 (5.7) |
| Liver | 6 (3.8) | 3 (5.9) | 3 (2.8) |
| Urinary | 4 (2.5) | 2 (3.9) | 2 (1.9) |
| Skeletomuscular | 2 (1.3) | 0 (0.0) | 2 (1.9) |
| Pancreas | 2 (1.3) | 1 (2.0) | 1 (0.9) |
| Eye | 1 (0.6) | 1 (2.0) | 0 (0.0) |
| General condition | 1 (0.6) | 0 (0.0) | 1 (0.9) |
| **Grade 3 and above irAEs** | **16 (10.2)** | **8 (15.7)** | **8 (7.5)** |
| Skin | 6 (3.8) | 3 (5.9) | 3 (2.8) |
| Respiratory | 4 (2.5) | 2 (3.9) | 2 (1.9) |
| Liver | 2 (1.3) | 2 (3.9) | 0 (0.0) |
| Skeletomuscular | 2 (1.3) | 0 (0.0) | 2 (1.9) |
| Pancreas | 2 (1.3) | 1 (2.0) | 1 (0.9) |
| Gastrointestinal | 1 (0.6) | 1 (2.0) | 0 (0.0) |

Variables were described as number (percentage, %).


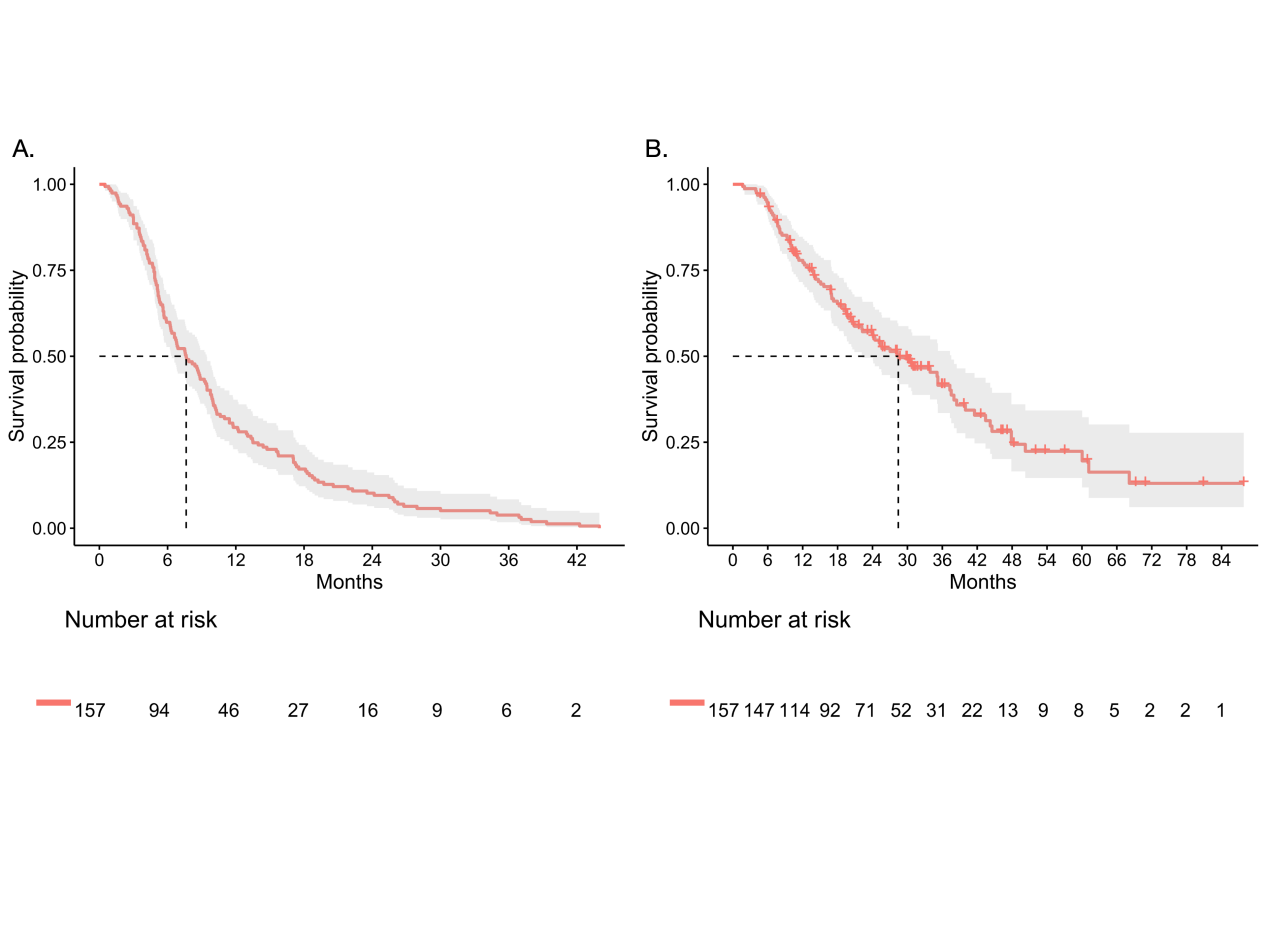


**Figure S1**. Survival of the total population during first-line immunotherapy.

1. PFS1 (median: 7.6 months, 95% CI: 6.4–9.4 months); B. OS (median: 28.4 months, 95% CI: 22.3–37.5 months).


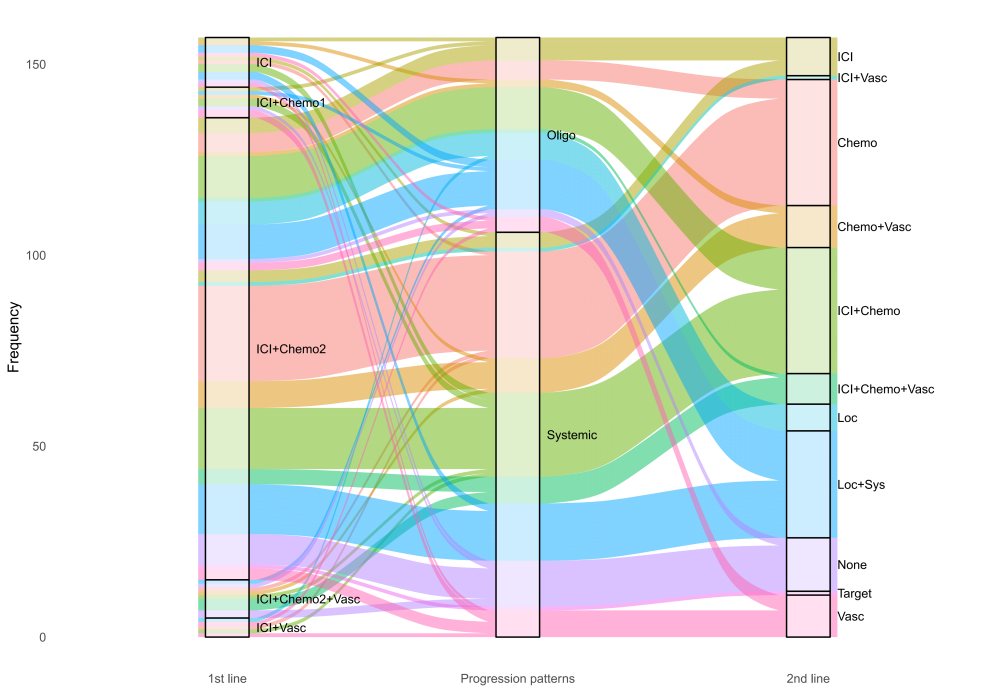


**Figure S2**. The Sankey diagram of first-line and second-line treatment regimens.

ICI, immune checkpoint inhibitors; Chemo1, single-agent chemotherapy; Chemo2, platinum doublet chemotherapy; Vasc, anti-vascular therapy; Oligo, oligoprogression; Systemic, systemic progression; Loc, local therapy; Sys, systemic therapy; Target, target therapy.
